# Generation of lung cancer cell lines harboring EGFR T790M mutation by CRISPR/Cas9-mediated genome editing

**DOI:** 10.18632/oncotarget.16752

**Published:** 2017-03-31

**Authors:** Mi-Young Park, Min Hee Jung, Eun Young Eo, Seokjoong Kim, Sang Hoon Lee, Yeon Joo Lee, Jong Sun Park, Young Jae Cho, Jin Haeng Chung, Cheol Hyeon Kim, Ho Il Yoon, Jae Ho Lee, Choon-Taek Lee

**Affiliations:** ^1^ Division of Pulmonology and Critical Care Medicine, Department of Internal Medicine and Respiratory Center, Seoul National University Bundang Hospital, Seongnam, Republic of Korea; ^2^ Toolgen Inc., Seoul, Republic of Korea; ^3^ Department of Internal Medicine, Seoul National University College of Medicine, Seoul, Republic of Korea; ^4^ Department of Pathology, Seoul National University Bundang Hospital, Seongnam, Republic of Korea; ^5^ Department of Internal Medicine, Korea Cancer Center Hospital, Seoul, Republic of Korea

**Keywords:** lung cancer, resistance, EGFR T790M, CRISPR/Cas9

## Abstract

Tyrosine kinase inhibitors (TKIs) such as gefitinib and erlotinib are effective against lung adenocarcinomas harboring epidermal growth factor receptor (EGFR) mutations. However, cancer cells can develop resistance to these agents with prolonged exposure; in over 50% of cases, this is attributable to the EGFR T790M mutation. Moreover, additional resistance mutations can arise with the use of new drugs. Cancer cell lines with specific mutations can enable the study of resistance mechanisms. In this study, we introduced the EGFR T790M mutation into the PC9 human lung cancer cell line—which has a deletion in exon 19 of the *EGFR* gene—by clustered regularly interspaced short palindromic repeats (CRISPR)/CRISPR-associated (Cas)9-mediated genome editing. EGFR pyrosequencing and peptide nucleic acid clamping revealed that PC9 cells with EGFR T790M generated by CRISPR/Cas 9 had a higher T790M mutation rate than those with the same mutation generated by long-term exposure to gefitinib (PC9-G); moreover, resistance to gefitinib in these clones was higher than that in PC9-G cells. The clones were also highly sensitive to the 3rd-generation EGFR TKI AZD9291, which is cytotoxic to lung cancer cells with EGFR T790M. The CRISPR/Cas9 programmable nuclease system can be used to generate various cancer cell lines with specific mutations that can facilitate studies on resistance mechanisms and drug efficacy.

## INTRODUCTION

The elucidation of oncogenic molecular mechanisms has changed lung cancer treatment approaches, especially for adenocarcinoma (ADCC). Mutations in epidermal growth factor receptor (EGFR) are important steps in ADCC development. A mutation in EGFR that confers sensitivity to EGFR tyrosine kinase inhibitor (TKI) has been identified in lung ADCC [1, 2]; accordingly, EGFR TKIs such as gefitinib or erlotinib have become standard treatment in cases of unresectable or metastatic ADCC with EGFR mutation. Although initially effective, most patients will experience disease progression due to the emergence of resistance to these drugs. Over half of TKI-resistant cases can be attributed to the EGFR-T790M mutation [3]. Therefore, new strategies for overcoming resistance are needed for lung cancer treatment.

Several 3rd-generation EGFR TKIs [e.g., AZD9291 (osimertinib) [4], CO1686 [5], and olmutinib [6] have been developed to overcome TKI resistance in lung cancer that have shown promising effects. However, new mutations such as EGFR C797S have emerged that show resistance to these 3rd-generation drugs [7–9]. To investigate the molecular mechanisms of resistance and devise effective counter strategies, it is necessary to establish cell lines that harbor the desired resistance mutations. These are typically generated by long-term continuous exposure of cancer cell lines to target drugs from very low to high concentrations. However, this process is lengthy—requiring several months or more—and there is no guarantee that the resistance mutation that arises will be of clinical significance.

In this study, we combined a CRISPR/Cas9-assisted precision genome editing technology derived from a bacterial adaptive immune system—clustered regularly interspaced short palindromic repeats (CRISPR)/CRISPR-associated (Cas)9—to generate a PC9 lung cancer cell line harboring EGFR T790M. The characteristics of this cell line were compared to those of PC9-G lung cancer cells with EGFR T790M generated by continuous exposure to gefitinib [10].

## RESULTS

### Point mutagenesis in human lung cancer cells using CRISPR/Cas9 ribonucleoprotein (RNP)

**Figure 1:**
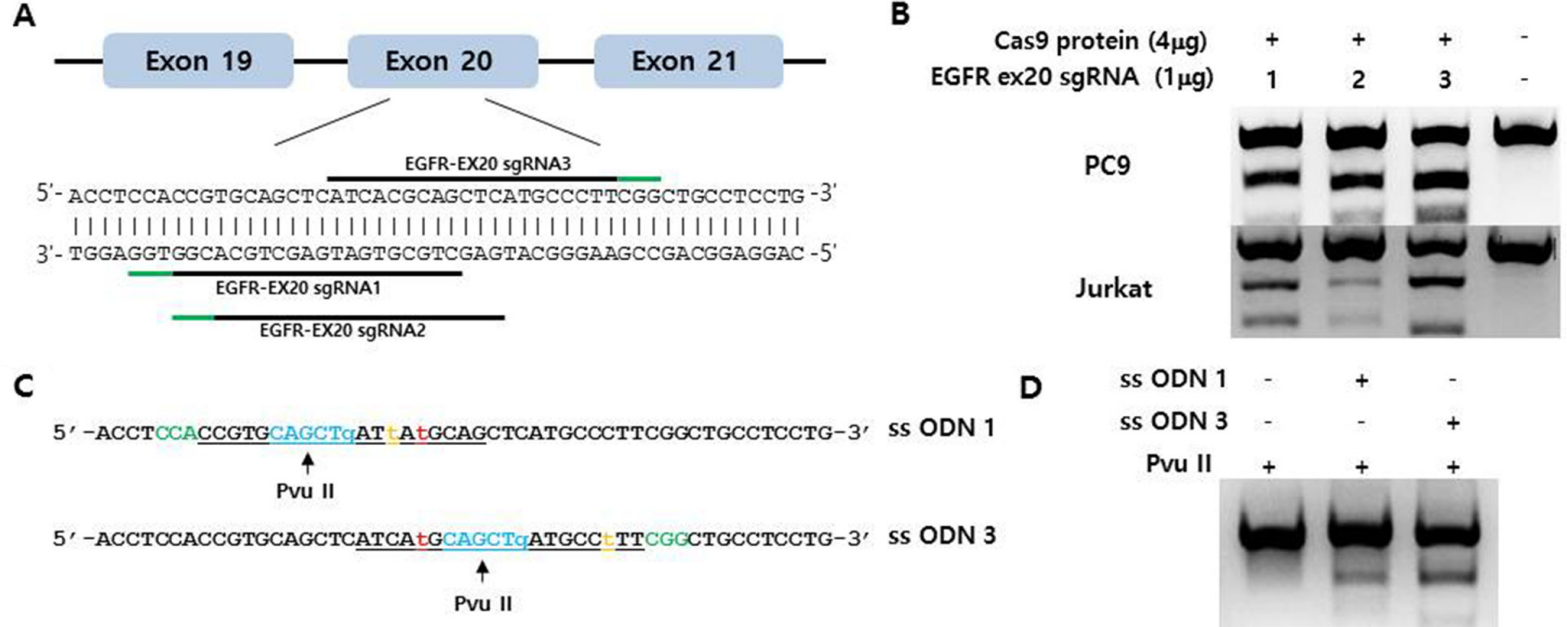
(**A**) Schematic representation of the CRISPR target site around the human *EGFR* T790 locus. The three tested target sequences are indicated by horizontal lines. Protospacer adjacent motifs (PAMs) are marked in green. (**B**) Indel mutation induced by EGFR-specific sgRNA/Cas9 protein electroporation into PC9 and Jurkat cells, as assessed by the T7E1 assay. (**C**) Single-stranded oligodeoxynucleotides (ssODNs) for specific knock-in of the T790M mutation (green, PAM sequences; orange, template mutation; blue, *Pvu*II restriction enzyme site; red, threonine 790 codon; underlined, sgRNA sequences). (**D**) Efficient homology-directed repair by CRISPR at the EGFR T790 locus. The ssODN donor along with the sgRNA/Cas9 complex was electroporated into PC9 cells. Specific knock-in alleles were assessed by *Pvu*II enzyme cleavage of the amplified EGFR T790M locus.

**Figure 2:**
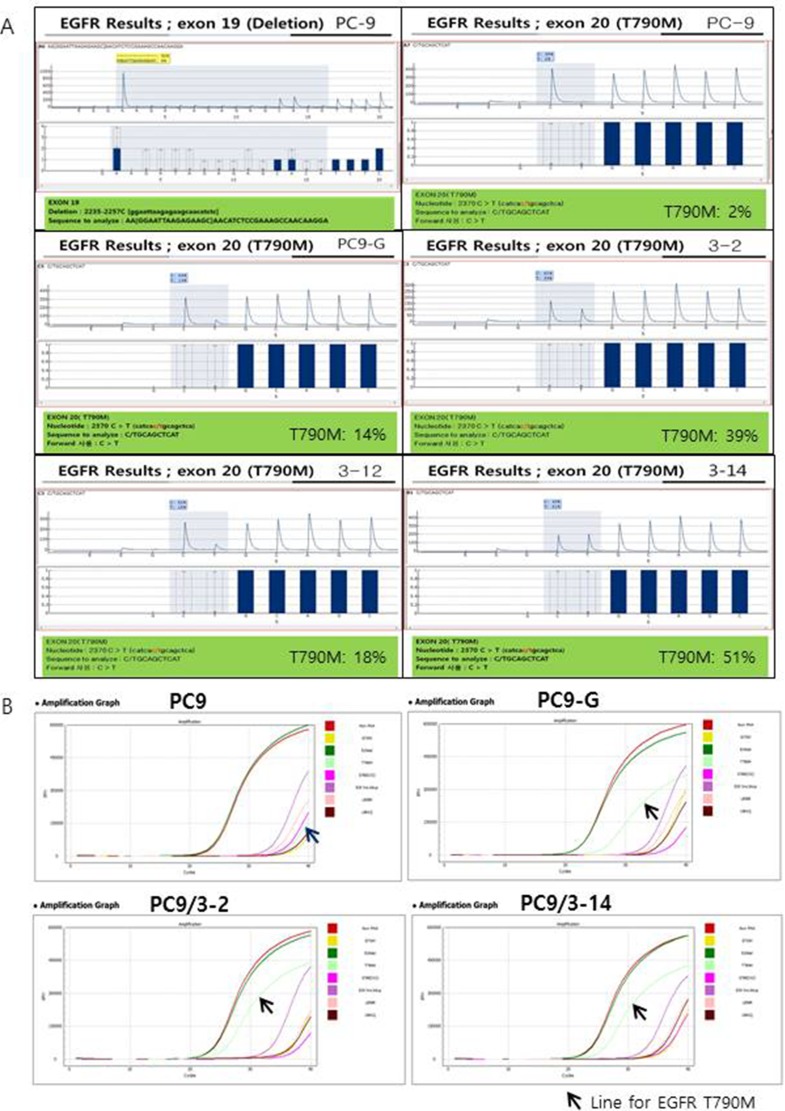
(**A**) Pyrosequencing for EGFR exon 19 and T790M. Exon 19 deletion (2235-2257C) was found in PC9 cells and all other cell lines tested (data not shown except for PC9). No T790M mutation was found in PC9 cells; however, high proportions of T790M mutations were found in PC9-G, PC9/3-2, PC9/3-12, and PC9/3-14 cells (14%, 39%, 18%, and 51%, respectively). (**B**) Peptide nucleic acid (PNA) clamping for EGFR exon 19 and T790M. ∆Ct-1 for E19del: 9.7 (PC9), 10.7 (PC9-G), 9.7 (PC9/3-2), and 9.8 (PC9/3-14): ∆Ct-1 for T790M: -3.5 (PC9), 6.2 (PC9-G), 7.7 (PC9/3-2), and 7.6 (PC9/3-14). The sample is considered to contain a mutation when ∆Ct-1 is more than 2.

We selected and tested three single-guided (sg) RNAs targeting the target sequence on exon 20 of *EGFR* or a nearby region in PC9 cells (Figure 1A). Three of these—i.e., sgRNA #1, #2, and #3—showed high genome editing efficiency of the target sequence in PC9 cells; however, sgRNA #2 was not effective in Jurkat cells (Figure 1B). We therefore designed single-stranded (ss)DNA donors to induce precise genome editing events upon co-delivery into cells with CRISPR RNP (Figure 1C). Together with the specific mutation (T790M), ssDNA donor also introduced a *Pvu*II restriction site, which was detectable in genomic DNA prepared from PC9 cells in which enhanced green fluorescent protein (EGFP) CRISPR RNP and ssDonor were introduced by electroporation (Figure 1D). When PC9 cells treated with EGFP CRISPR RNP and ssDonor were cultured in the presence of gefitinib at a concentration that was toxic to untreated PC9 cells, many surviving PC9 clones were obtained. The EGFP CRISPR/RNP target sites of several gefitinib-resistant clones were analyzed by pyrosequencing of genomic DNA and peptide nucleic acid (PNA) clamping (Figure 2). The parent cell line showed a rate of T790M mutation in exon 20 of *EGFR* of 2%, which is the lower limit of detection by pyrosequencing. In contrast, PC9-G cells generated by long-term exposure to gefitinib showed a T790M mutation rate of 14%. On the other hand, PC9/3-2 and PC9/3-14 cells (i.e., 2nd and 14th clones isolated from sgRNA 3, respectively) showed mutation rates of 39% and 51%, respectively. We also tested four clones from sgRNA1; however, none had a T790M mutation rate > 5%.

We confirmed the presence of EGFR T790M by PNA clamping. The change in threshold cycle (∆Ct)-1 values for T790M were −3.5 (PC9), 6.2 (PC9-G), 7.7 (PC9/3-2), and 7.6 (PC9/3-14). ∆Ct-1 > 2 indicated the presence of a mutation. The EGFR T790M mutation rates in PC9/3-2 and 3-14 were higher than that in PC9-G. Thus, PC9/3-2 and PC9/3-14 cells developed by precise genome editing technology had a higher content of the T790M mutant allele than PC9-G cells, although a minor discrepancy in the T790M mutation rate obtained by pyrosequencing and PNA clamping methods were observed in PC9/3-2 and PC/3-14 cells.

### EGFR protein expression levels in mutated human lung cancer cell lines

**Figure 3:**
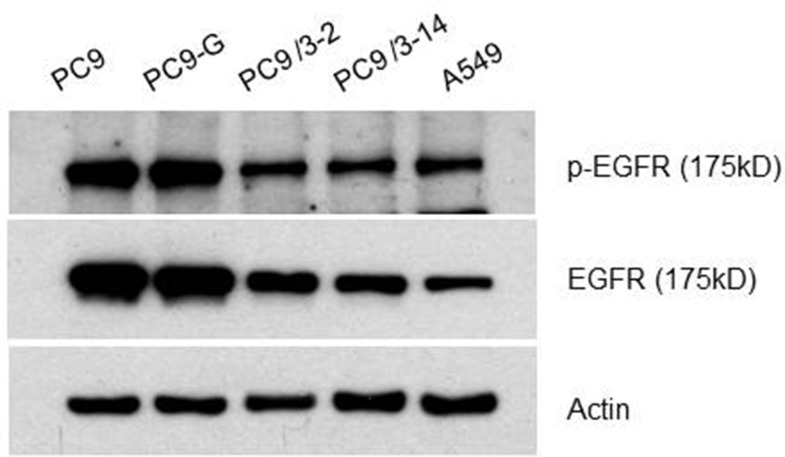
Western blot analysis for the EGFR protein All EGFR proteins from PC9, PC9-G, PC9/3-2,-14 and A549 were of the same size.

EGFR and phosphorylated (p)EGFR protein levels in mutated human lung cancer cell lines were analyzed by western blotting (Figure 3). The levels of both proteins were slightly lower in PC9/3-2 and PC9/3-14 cells than in PC9, PC9-G, and A549 cells, although the EGFR to pEGFR ratio was similar among cell lines.

### Reduced sensitivity of mutated human lung cancer cell lines to gefitinib

**Figure 4:**
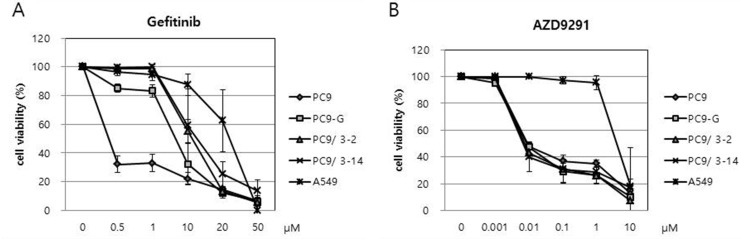
(**A**) Reduced sensitivities of PC9-G, PC9/3-2, and PC9/3-14 cells to gefitinib compared to that of PC9 cells. PC9/3-2 and PC9/3-14 cells were more resistant to gefitinib than PC9-G cells. IC50: PC9 vs PC9-G, PC9/3-2, PC9/3-14 *p* < 0.01; PC9-G vs PC9/3-2, PC9/3-14 *p* < 0.05; PC9/3-2 vs PC9/3-14, not significant, (**B**) PC9, PC9-G, PC9/3-2 and PC9/3.14 were similarly sensitive to AZD9291, a 3rd generation EGFR tyrosine kinase inhibitor. IC50: PC9 vs PC9-G vs PC9/3-2 vs PC9/3-14, not significant.

We analyzed the sensitivity of PC9/3-2 and PC9/3-14 cells relative to that of PC9, PC9-G, and A549 cells to gefitinib with the 3-(4,5-dimethylthiazol-2-yl)-5-(3-carboxymethoxyphenyl)-2-(4-sulfophenyl)-2H-tetrazolium salt (MTS) assay. Cells were treated with gefitinib at concentrations ranging from 0.5 to 50 μM, and proliferation was evaluated 96 h later. PC9 cells were the most sensitive to gefitinib, whereas PC9-G cells showed greater resistance. Interestingly, PC9/3-2 and PC9/3-14 cells showed higher resistance than the parental PC9 cell line and PC9-G cells. The two cell lines did not differ in terms of gefitinib resistance. A549 cells harboring wild-type EGFR showed the greatest resistance to gefitinib (Figure 4A and Table 1).

**Table 1:** Summary of EGFR T790M mutation rate and half-maximal inhibitory concentration (IC50) to gefitinib and AZD9291 of each cell line

	PC9	PC9-G	PC9/3-2	PC9/3-14	A549
Rate of EGFR T790M					
Pyrosequencing	2%	14%	39%	51%	
PNA clamping (∆Ct-1)	−3.5	6.2	7.7	7.6	
IC50 (μM) to gefitinib*	0.37 ± 0.033	7.21 ± 1.72	11.59 ± 1.27	12.34 ± 4.76	24.85 ± 7.12
IC50 (nM) to AZD9291**	11.2 ± 3.2	12.3 ± 1.4	8.9 ± 0.5	14.8 ± 12.1	6884 ± 2913

^*^ IC50 to gefitinib: PC9 vs. PC9-G, PC9/3-2, and PC9/3-14; *P* < 0.01, PC9-G vs. PC9/3-2 and PC9/3-14; *P* < 0.05, PC9/3-2 vs. PC9/3-14 (not significant by the *t* test).

^**^ IC50 to AZD9291: PC9 vs. PC9-G vs. PC9/3-2 vs. PC9/3-14 (not significant).

### Human lung cancer cell lines harboring EGFR T790M mutation are inhibited by a 3rd-generation EGFR TKI

We tested the viability of human lung cancer cell lines upon treatment with the 3rd-generation EGFR TKI AZD9291 (osimertinib), which has been shown to be cytotoxic to lung cancer cells harboring the EGFR T790M mutation. Cells were treated with various doses of AZD9291 and viability was assessed 96 h later with the MTS assay (Figure 4B). Interestingly, the sensitivities of PC9-G, PC9/3-2, and PC9/3-14 cell lines to AZD9291 were similar to that of PC9 cells (Table 1). In contrast, A549 cells were highly resistant to AZD9291 relative to the other cell lines. These data indicate that human lung cancer cell lines harboring EGFR T790M mutation engineered by CRISPR/Cas9 can be inhibited by the 3rd-generation EGFR TKI AZD9291.

### PC9 cells harboring EGFR T790M show increased apoptosis upon treatment with AZD9291 but not gefitinib

**Figure 5:**
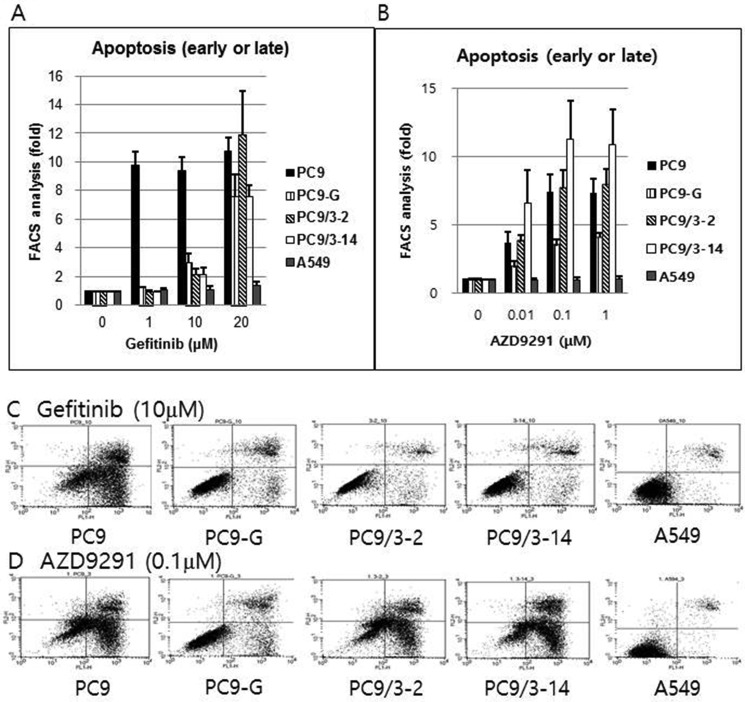
Proportion of early or late apoptotic cell death after gefitinib (**A**) or AZD9291 (**B**) treatment, as measured by flow cytometry after staining with Annexin V and propidium iodide. Gefitinib treatment failed to induce significant apoptosis in PC9-G, PC9/3-2, and PC9/3-14 cells up to a concentration of 10 μM. However, AZD9291 treatment showed strong apoptosis induction in PC9-G, PC9/3-2, and PC9/3-14 cells, similar to that observed in PC9 cells. A549 cells (harboring wild type EGFR) were not affected by gefitinib or AZD9291 treatment. (fold increase in the proportion of apoptotic cells compared to that in control treated with 0 μM concentration) Actual flow cytometry data of early or late apoptotic cell death after gefitinib (10 μM) (**C**) or AZD9291 (0.1 μM) treatment (**D**) after staining with Annexin V and propidium iodide. Gefitinib treatment failed to induce significant apoptosis in PC9-G, PC9/3-2, and PC9/3-14 cells at 10 μM. However, AZD9291 treatment showed strong apoptosis induction in PC9-G, PC9/3-2, and PC9/3-14, cells similar to that observed in PC9 cells. A549 cells (harboring wild type EGFR) were not affected by gefitinib or AZD9291 treatment.

The sensitivity of lung cancer cell lines to gefitinib and AZD9291 was investigated by detecting apoptotic cells labeled with Annexin V. Gefitinib did not induce significant apoptosis in PC9-G, PC9/3-2, or PC9/3-14 cells at concentrations up to 10 μM. However, AZD9291 potently induced apoptosis in the three cell lines at rates similar to that in PC9 cells. In contrast, A549 cells harboring wild-type EGFR was unaffected by gefitinib and AZD9291 treatment (Figure 5).

## DISCUSSION

In cases where several different mutations are present in patients who exhibit TKI resistance, it is almost impossible to generate cell lines that harbor the specific mutation using the traditional method of exposing cells to increasing concentrations of drug. Inducing specific mutations in cell lines by genetic approaches can facilitate the development of new and effective drugs. CRISPR/Cas9 nuclease is one example of engineered nuclease platforms that can be used for this purpose [11]. In this study, we used CRISPR/Cas9 nucleases-assisted genome editing to generate lung cancer cell lines harboring the EGFR T790M mutation. An advantage of this system is that generating an endonuclease is easier than by other methods; with a constant Cas9 component, only a guide RNA with a 20-bp sequence complementary to a new target site needs to be generated by simple molecular cloning to obtain an expression vector, with enzymatic or chemical production of short RNA molecules [12, 13]. Of the three sgRNA sequences we initially designed for target site in the EGFR gene, two were used successfully to establish PC9 cell clones with the T790M mutations that exhibited gefitinib resistance. Pyrosequencing of the EGFR gene revealed that three clones derived from sgRNA3 (3-2, 3-12, and 3-14) had high rates of T790M mutation (39%, 18%, and 51%, respectively) while retaining the exon 19 deletion (2234–2257C) of the parental cell line (PC-9) (Figure 2A). The PNA clamping assay showed similar results, except that a discrepancy was found in the rate of T790M mutation obtained by the pyrosequencing and PNA clamping approaches in PC9/3-12 and 3/14 cells (Figure 2B). Two of the clones (3-2 and 3-14) showed stronger resistance to gefitinib than PC9-G cells, and there was negligible apoptosis induced in these lines even at concentrations as high as 10 μM, suggesting that the degree of resistance is related to the rate of T790M mutation. On the other hand, PC9-3/2 and 3/14 cell lines were highly sensitive to AZD9291, a 3rd-generation TKI, indicating that these cells exhibit clinically relevant responses to drug treatment.

In conclusion, we generated lung cancer cell lines harboring an EGFR T790M mutation with a CRISPR/Cas9-based approach. This technique can be used to generate cell lines with different mutations of clinical significance such as EGFR C797S, which can facilitate the study of resistance mechanisms as well as drug testing.

## MATERIALS AND METHODS

### Cell lines and culture

PC-9 is lung adenocarcinoma cell line with a deletion in exon 19 of the *EGFR* gene that exhibits high sensitivity to TKIs. The PC-9G cell line (PC-9 cells resistant to gefitinib) was established by long-term exposure to gefitinib, and was shown to harbor the EGFR T790M mutation [10]. The cell lines were a gift from Dr. C.H. Kim (Korea Cancer Center Hospital). A549 cells harboring wild-type EGFR were purchased from the American Type Culture Collection (Manassas, VA, USA). Cells were maintained in Roswell Park Memorial Institute (RPMI) 1640 medium containing 10% fetal bovine serum and 1% penicillin/streptomycin.

### Antibodies and EGFR TKI

The antibody against EGFR was purchased from Abcam (Cambridge, UK; ab2430) and the antibody against actin was purchased from Santa Cruz Biotechnology (Santa Cruz, CA, USA; I-19). Gefitinib was obtained from Cell Signaling Technology (Danvers, MA, USA) and AZD9291 (osimertinib) was from Selleckchem (Houston, TX, USA).

### Generation of PC9 cell lines with EGFR T790M mutation by CRISPR/Cas9 nucleases

#### Preparation of Cas9 protein with sgRNAs and single-stranded oligodeoxynucleotides (ssODNs)

Recombinant Cas9 protein tagged with a nuclear localization signal and a his6 tag were expressed in *E. coli* (BL21-DE3 strain) and purified by Ni-nitrilotriacetic acid chromatography. Three sgRNAs were synthesized by *in vitro* transcription using T7 RNA polymerase as previously described [13] (Figure 1A). The oligonucleotide used for homology-directed recombination consisted of a 127-base ssODN molecule containing two 50-bp outer arms homologous to the endogenous *EGFR* genomic region flanking the exon encoding T790, and was synthesized by Integrated DNA Technologies (San Diego, CA, USA).

### Electroporation, genotyping, and establishment of gefitinib-resistant PC9 cell lines

CRISPR RNPs and ssODN donors were electroporated into Jurkat cells (two 20-ms pulses at 1300 V) and PC9 cells (single 30-ms pulse at 950 V) using the Neon transfection system (Invitrogen) The T7E1 assay was performed as previously described (Figure 1B) [14]. To establish gefitinib-resistant PC9 cell lines, cells were transfected with EGFR CRISPR RNPs along with the ssODN donor and cultured for 7 days to allow recovery. Gefitinib was then added to the medium at a final concentration of 1 μM for 2 weeks. Surviving cells were seeded in 96-well plates at a density of 0.5 cells per well in order to establish a monoclonal cell population. Genomic DNA from isolated monoclonal gefitinib-resistant clones was subjected to pyrosequencing to confirm the presence of edited EGFR alleles.

### *EGFR* pyrosequencing

*EGFR* mutations in exons 18–21 were detected by pyrosequencing as previously described [15, 16]. A 40-μl aliquot of PCR product was bound to streptavidin Sepharose High Performance (GE Healthcare, Uppsala, Sweden), purified, washed, denatured in 0.2 mol/l NaOH solution, and washed again. The pyrosequencing primer (0.3 μmol/l) was then annealed to the purified single-stranded PCR product, and pyrosequencing was performed on a PyroMark ID system (Qiagen, Valencia, CA, USA) according to the manufacturer's instructions.

### PNA clamping method for *EGFR* exon 19 deletion and generation of T790M mutation

The PNA Clamp EGFR Mutation Detection kit (Panagene, Daejeon, Korea) was used to detect *EGFR* gene mutations by real-time PCR. All reactions were performed in a 20-μl volume with template DNA, primer and PNA probe sets, and fluorophore PCR master mix. Real-time PCR was performed using a CFX 96 kit (Bio-Rad, Philadelphia, PA, USA). PCR cycling conditions were as follows: 94°C for 5 min, followed by 40 cycles of 94°C for 30 s, 70°C for 20 s, 63°C for 30 s, and 72°C for 30 s. Each of 29 *EGFR* gene mutations was detected by one-step PNA-mediated real-time PCR clamping. PCR efficiency was determined by measuring Ct values, which were automatically calculated from PCR amplification plots of fluorescence vs. number of cycles. ∆Ct-1 was calculated to ensure that the sample and standard Ct values were from the tested and wild-type DNA control samples, respectively. The sample was considered to harbor a mutation when the ∆Ct-1 value (standard minus sample Ct value) was > 2.0 [17].

### Western blot analysis of EGFR expression

Cell lysates (40 μg) were analyzed by western blotting using an anti-EGFR and anti-phosphorylated EGFR antibody. Actin was used as a loading control.

### Cell proliferation assay

PC9, PC9-G, PC9-3-2, PC9-3-14, and A549 human lung cancer cell lines were seeded at 3 × 10^3^ cells/well in 96-well plates and treated with gefitinib or AZD9291; 96 h later, cell proliferation was assessed using the CellTiter 96 AQueous One Solution Cell Proliferation Assay kit (Promega, Madison, WI, USA) according to the manufacturer's instructions. Three independent experiments were performed and used to determine mean half-maximal inhibitory concentration (IC50).

### Apoptosis assay

Human lung cancer cell lines were treated with gefitinib or AZD9291 for 48 h, and apoptotic cells were detected using the FITC Annexin V Apoptosis Detection kit (BD Pharmingen, San Diego, CA, USA) according to the manufacturer's protocol on a FACSCalibur flow cytometer (Becton Dickinson, San Jose, CA, USA). Three independent experiments were performed and the mean proportions of cells in early or late apoptosis were determined.
